# Swimming Attenuates Muscle Wasting and Mediates Multiple Signaling Pathways and Metabolites in CT-26 Bearing Mice

**DOI:** 10.3389/fmolb.2021.812681

**Published:** 2022-01-17

**Authors:** Jiapeng Li, Qiurong Xie, Liya Liu, Ying Cheng, Yuying Han, Xiaoping Chen, Jia Lin, Zuanfang Li, Huixin Liu, Xiuli Zhang, Haichun Chen, Jun Peng, Aling Shen

**Affiliations:** ^1^ The Department of Physical Education, Fujian University of Traditional Chinese Medicine, Fuzhou, China; ^2^ School of Physical Education and Sport Science, Fujian Normal University, Fuzhou, China; ^3^ Academy of Integrative Medicine, Fuzhou, China; ^4^ Fujian Key Laboratory of Integrative Medicine in Geriatrics, Fuzhou, China; ^5^ Rehabilitation Industry Institute, Fujian University of Traditional Chinese Medicine, Fuzhou, China; ^6^ Provincial University Key Laboratory of Sport and Health Science, School of Physical Education and Sport Sciences, Fujian Normal University, Fuzhou, China

**Keywords:** swimming, colorectal cancer, muscle wasting, NF-κB, metabolite

## Abstract

**Objectives:** To investigate the effects of swimming on cancer induced muscle wasting and explore its underlying mechanism in CT-26 bearing mice.

**Methods:** BALB/c mice (*n* = 16) injected with CT-26 cells were divided into two groups, including Tumor group (*n* = 8) and Swimming group (*n* = 8). Another 8 un-injected mice were set as Control group. Mice in Swimming group were subjected to physical training for swimming twice per day for 30 min intervals and 6 days per week for a total of 4 weeks. The tumor volume was monitored every 3 days and tumor weight was measured at the end of experiment. The changes of muscle function, pathological and cell apoptosis of quadriceps muscles were further assessed, and its underlying mechanisms were further explored using multiple biological technologies.

**Results:** Swimming obviously alleviated tumor volume and weight in CT-26 bearing mice. Moreover, swimming attenuated the decrease of muscle tension, autonomic activities, and increase of muscle atrophy, pathological ultrastructure, as well as cell apoptosis of quadriceps muscles in CT-26 bearing mice. Furthermore, swimming significantly down-regulated the protein expression of NF-κB, p-NF-κB, TNF-α, IL-1β, IL-6 and Bax, while up-regulated the expression of Bcl-2. Further differential expressed metabolites (DEMs) analysis identified a total of 76 (in anion mode) and 330 (in cationic mode) DEMs in quadriceps muscles of CT-26 bearing mice after swimming, including taurochenodeoxycholic acid, taurocholic acid, ascorbic acid and eicosapentaenoic acid.

**Conclusion:** Swimming attenuates tumor growth and muscle wasting, and by suppressing the activation of NF-κB signaling pathway mediated inflammation, reducing the level of Bax medicated cell apoptosis, as well as modulating multiple metabolites might be the importantly underlying mechanisms.

## Introduction

Cancer has become a leading cause of death worldwide, including in China, with an increasing burden of cancer incidence and mortality observed over the past half century (Wei et al., 2020). Recently statistics indicated that there are 18.1 million new cases and 9.6 million cancer deaths worldwide in 2018, of which, nearly 24% (4.3 million) of these cancer cases and 30% (2.9 million) of deaths have occurred in China (Sung et al., 2021). As the population ages, the number of cases of cancer will continue to rise significantly (Chen et al., 2016). Many cancers trigger rapid muscle wasting, a condition also known as cachexia, that in turn leads to resistance to treatment, low quality of life and death (Baracos et al., 2018). Though couples of therapeutic strategies have been adopted in an attempt to alleviate cancer cachexia and improve the quality of life of cancer patients (Kumar et al., 2010). Due to the complex processes of cancer induced cachexia, most of these therapeutic approaches based on a single target, have failed so far. Therefore, efforts to further explore the strategies on attenuating cancer induced cachexia may ultimately improve treatment response and quality of life of cancer patients.

As one of the primary causes of morbidity and mortality associated with cancer, cachexia is most commonly observed in individuals with advanced cancer, presenting in 80% of these patients (Fukawa et al., 2016). One of prominent clinical features of cancer-induced cachexia is muscle wasting, which is a complex phenomenon and characterized by significant decrease in muscle fiber cross-sectional area, myonuclear number, protein content and muscle strength (Musolino et al., 2016). These structural and functional changes in muscles deteriorate the quality of life and even lead to human disease and death (Dutt et al., 2018).

The pathophysiologic mechanisms underlying muscle wasting in cachexia are complex, including systemic inflammation (Aversa et al., 2017). Multiple cytokines, including TNF-α, interleukin-1 (IL-1), IL-6 and IFN-γ, had been reported to play an essential role in the induction of cancer-related muscle wasting (Argiles and Lopez-Soriano, 1999). Increasing studies revealed that the elevated levels of proinflammatory cytokine TNF-α in patients play an important role in the evolvement of muscle wasting (Jekell et al., 2004) *via* inducing the breakdown of mature myotubes (Guttridge et al., 2000). With the stimulation of TNF-α, the activation of transcription factor NF-κB is obviously increased, which in turn promotes muscle wasting by inhibiting the synthesis of MyoD and muscle regeneration, as well as promoting atrophy (Bowen et al., 2015). The activation of NF-κB further promotes the synthesis of cytokines, which can contribute to muscle wasting as described above (Thoma and Lightfoot, 2018).

These studies suggested that inhibiting cancer induced inflammation by reducing the levels of pro-inflammation cytokines and activation of NF-κB pathway represent a strategy for treatment of cancer induced muscle wasting. Recently both epidemiological studies and clinical trials revealed that the prognosis of physically active cancer patients is improved due to the exercise performed after cancer diagnosis as opposed to exercise habits before the disease (Irwin et al., 2008). Since exercise strongly related to skeletal muscle, exercise training has long been proposed to counteract muscle wasting in cachexia (Zhang et al., 2019). Proper physical exercise increases muscle mass and volume, enhances immune system function and improves metabolism and body composition (Re Cecconi et al., 2019).

Due to the difficulty for cancer patients to perform high-intensity exercises, low-intensity exercises, like swimming might be more acceptable. However, as a major kind of aquatic exercise, the benefits of swimming and its underlying mechanisms on cancer induced muscle wasting remains largely unknown. In the current study, we evaluated the benefits of swimming on tumor growth, muscle wasting, and its underlying mechanisms.

## Materials and Methods

### Reagent and Antibodies

Fetal bovine serum (cat. no. 10099141), RPMI 1640 (cat. no. 1049101), Trypsin-EDTA (cat. no. 25200072), penicillin-streptomycin (cat. no. sv30010) and the BCA protein assay kit (cat. no. 23225) were purchased from Thermo Fisher Scientific (Sunnyvale, CA, USA). Antibodies against interleukin-6 (IL-6) (cat no. ab208113), NF-κB (cat. no. Abm40053), p-NF-κB (cat. no. Abm50373) and tubulin (cat. no.11H10) were purchased from Abcam (Cambridge, United Kingdom), while interleukin-1β (IL-1β) (cat. no. 12242S), Bax (cat. no.14796), Bcl-2 (cat. no. 2876) antibodies and Horseradish peroxidase (HRP)-conjugated secondary antibody (cat. no.7074) were bought from Cell Signaling Technology (Danvers, MA, USA). TNF-α (cat. no. 41504) antibody was obtained from SAB (MD, USA). Hematoxylin solution (cat. no. G1140) and eosin solution (cat. no. G1100) were purchased from Solarbio (Beijing, China). RIPA lysis buffer (cat. no. P0013, Beyotime, Shanghai, China), protease inhibitor (cat. no. 539131-10VLCN, MCE, NJ, USA) and phosphatase inhibitors (cat. no. 4906845001, Roche, Basel, Switzerland) were used for protein extraction. Matrigel (cat. no. 354234) was purchased from BD Biosciences (San Jose, CA, USA).

### Cell Culture

The CT-26 murine colon carcinoma cell line was purchased from the Shanghai Cell Bank of the Chinese Academy of Sciences (Shanghai, China). Cells were cultured in RPMI-1640 medium (cat. no. 8121369) supplemented with 10% of FBS (cat. no. 2254375CP) and 1% of penicillin (100 U/ml) and streptomycin (100 μg/ml) in a 37°C, 5% of CO_2_, humidify of incubator. Cells were sub-cultured at 80–90% of confluence.

### Animal Experiments

Male BALB/c mice (5–6 weeks; 20.8 ± 1.4 g) were purchased from Shanghai SLAC Laboratory Animal Co. (Shanghai, China) and acclimatized for 1 week before used. Mice were housed under a specific pathogen-free (SPF) condition with a 24–28°C of temperature, 60 ± 5% of humidity, 12-h dark/light cycle. Food and water were given ad libitum throughout the experiment. Animal care and experiments were performed in strictly accordance with the “Guide for the Care and Use of Laboratory Animals” and the “Principles for the Utilization and Care of Vertebrate Animals”, and approved by the Committee of Fujian University of Traditional Chinese Medicine (No. FJTCM IACUC 2019042).

Construction of mouse Xenograft.—After the acclimation period, CT-26 cells (1 × 10^6^ cells/ml) in a total volume of 100 μl of PBS containing 50% matrigel were injected subcutaneously into the right flank area of the mice (*n* = 16). After seeding of 3 days, the tumor-bearing mice were randomly divided into Tumor group (*n* = 8) and Swimming group (*n* = 8) according to tumor volume. Another 8 un-injected mice were set as Control group.

Swimming training—In the current study, CT-26 bearing mice in the swimming group were subjected to physical training in water (30 ± 2°C) twice per day for 30 min intervals. This training was conducted 6 days per week for a total of 4 weeks. The mice participated in voluntarily swim for an estimated 10 min at initially, then floated on the water, and swam intermittently, each time extended for 10 min until 30 min. In order to make the mice swim continuously for longer duration, we used a stick to pull the water to drive them.

### Tumor Volume and Weight

Measurement of tumor volume and tumor weight— During the experiment, the electronic vernier caliper was used to measure the major (L) and minor (W) diameter of tumors. The tumor volume was calculated every 3 days on the basis of the following formula: tumor volume = L×W^2^/2. At the end of experiment, the mice were anaesthetized with isoflurane and tumor tissues were removed and weighed, followed by fixed with 4% of formaldehyde until used.

### Grip Force Assessment and Autonomic Activity Test

Skeletal muscular tension of mice was quantified by the grip-strength test. The grip-tension device (cat. no. DS2-20N) was obtained from Xinruan Corp. (Shanghai, China) and was comprised of a mesh tension test board connected to an isometric force transducer. Basically, the grip-tension meter was positioned horizontally, and the mice were held by the tail and lowered towards the device. The mice were allowed to grasp the mesh tension test board and pulled backwards in the horizontal plane. The force applied to the bar just before it lost grip was recorded as the peak tension and carried out five times for each mouse. The strength test was performed before the first training, after the 2nd and 4th week of training.

Autonomic activity of mice was measured by the multifunctional mice independent activities recorder (cat. no. ZZ6, Taimeng Corp, Chengdu, China). The mice were placed in a dark compartment and covered with a light-shielding lid. The mice activities recorder with 36 infrared sensing points was used to count the number of mice that stood continuously within 30 min.

### HE Staining

The cross-sectional tissues of quadriceps femoris from each mouse were fixed with 4% paraformaldehyde (PH 7.4) for 24 h, processed, embedded in paraffin, and cut into 4-μm-thick sections. The sections were dewaxed and dehydrated. For histologic assessments, sections were stained with hematoxylin solution for 60 s, differentiated with 1% hydrochloric acid ethanol for 3 s, and stained with eosin-phloxine solution for 20 s. The tissues on each slide were added to a coverslip and imaged at magnification of 200 using a Leica DM400B microscope (Leica, Wetzlar, Germany).

### Transmission Electron Microscopy Analysis

Tissue pieces of quadriceps muscle (about 1 mm^3^) from each mouse were immersion-fixed for 2 h at 4°C in 2.5% glutaraldehyde and 1% paraformaldehyde in 0.2 M phosphate buffer (pH 7.4), washed, and then postfixed in 1% osmium tetroxide. After rinsing in the phosphate buffer, the tissue pieces were dehydrated in ascending grades of ethanol ending with propylene oxide and embedded in epoxy resin. Semithin sections (1 μm) were prepared using ultra microtome (Leica, Germany) and stained with 1% toluidine blue. Ultrathin sections (90 nm) were cut, mounted on copper grids and stained with 2% uranyl acetate for 10 min, followed by lead citrate staining for 10 min and examined in a transmission electron microscope (Hitachi H-7650, Hitachi High-Technologies Corporation. Japan).

### TUNEL Staining of Muscle Cells

TUNEL staining was performed to detect muscle cell apoptosis, according to the instructions. After dewaxing and gradient alcohol, the sections were incubated in proteinase K working solution at 37°C in a humidified atmosphere for 15 min. TUNEL reaction mixture (50 μl) was added and incubated for 60 min at 37°C. After rinsed with PBS for 3 times, 50 μl of converter-peroxidase was added to the sections and incubated at 37°C for an additional 30 min, and then rinsed with PBS for three times, incubated with the 100 μl diaminobenzidine substrate. Counterstained with hematoxylin and analyzed by light microscopy (Leica DM400B, Germany) at ×400 magnification. The cells with brown nucleus were defined as apoptotic cells. The percentage of apoptotic cells was calculated as the ratio of the number of TUNEL-positive cells to the total number of cells.

### Immunohistochemical Analysis

The sections from each group were subjected to antigen retrieval and the endogenous peroxidase activity, and blocked with 3% hydrogen peroxide. After blocking non-specific proteins at room temperature for 10 min, the sections were incubated with primary antibodies against Bax, Bcl-2, TNF-α, IL-1β, IL-6, NF-κB or p-NF-κB (all in dilution, 1:200) at 4°C overnight. After washed with PBS, the slides were incubated with HRP-conjugated secondary antibody and then washed with PBS. The sections were then incubated with DAB as the chromogen, followed by counterstaining with diluted hematoxylin. After staining, five randomly selected images from each sample were taken at a magnification of ×400, and the average percentage of positive stained cells in each field was counted using Image-Pro Plus (Media Cybernetics, Rockville, MD, USA).

### Analysis of Western-Blotting

The muscle tissues were homogenized and total protein were extracted using RIPA lysis buffer, and centrifuged for 10 min (12,000 rpm, 4°C), the supernatant with total protein was aspirated into a new clean tube. The BCA Protein Assay Kit was used to determine protein concentrations according to the manufacturer’s protocol. Equal amounts of total protein from each sample were separated by 10% sodium dodecyl sulfate (SDS)-polyacrylamidegel electrophoresis (PAGE), transferred to polyvinylidene fluoride (PVDF) membrane (cat. no. ISEQ00010, Millipore, MA, USA). Non-specific protein interactions were blocked by incubation with 5% non-fat milk in Tris-buffered saline with Tween 20 (50 mM Tris-HCl, 150 mM NaCl and 0.05% Tween 20; pH 7.6) at room temperature for 2 h. Membranes were incubated with primary antibodies against Bcl-2, Bax or p-NF-κB (all in 1:1,000 dilution) overnight at 4°C followed by incubation with an HRP-conjugated secondary antibody (1:5,000 dilution) at room temperature for 2 h. Proteins were detected using a chemiluminescence detection system (Bio-Rad Laboratories, Inc., Hercules, CA, USA) and were visualized using enhanced chemiluminescence reagent (Beyotime, Shanghai, China). Tubulin was used as internal control.

### Metabolomics Study

Quadricep muscles of mice from both Tumor group (*n* = 5) and the Swimming groups (*n* = 5) were collected and stored at −80°C. For metabolic profiling, archived quadriceps muscle samples were ground and mixed with 1:10 (w/v) ice cold extraction solution (a mixture of water, methanol, acetonitrile acentone with the volume ratio of 1:3:3:3 respectively). The mixtures were shaken at room temperature for 10 min, and then placed in the refrigerator at -20°C for 30 min, followed by centrifugation of the mixtures for 15 min (14,000 g, 4°C). The supernatant fractions were collected for LC-MS analysis. A pooled quality control (QC) sample was prepared by mixing equal amounts of each quadricep muscle sample. LC-MS analysis was performed on Atlantis PREMIER BEH C18 AX VanGuard FIT Column using the UHPLC 3000 System coupled to a Q Exactive System (Thermo Fisher Scientific), at both positive and negative ion modes. The metabolites were identified by MZCloud and ChemSpider. The MetaboAnalst (http://www.metaboanalyst) was used to data analysis and data visualization, including PCA (Principal Component Analysis), Cluster heat map, Metabolite Collection and Enrichment Analysis (MCEA), and Metabolite Pathway Analysis (METPA).

### Statistical Analysis

Statistical analyses were performed using the SPSS statistical program (SPSS/PC+, version 22.0, Chicago, IL, USA). The results were presented as the mean value ±standard deviation (SD). Three group data used One-way analysis of variance (ANOVA) to compare statistical significance when the data met the normal distribution. Two group data used Student’s T-TEST. Differences associated with *p* < 0.05 were considered statistically significant.

## Results

### Swimming Attenuates Tumor Growth of CT-26 Cells *In Vivo*

**FIGURE 1:**
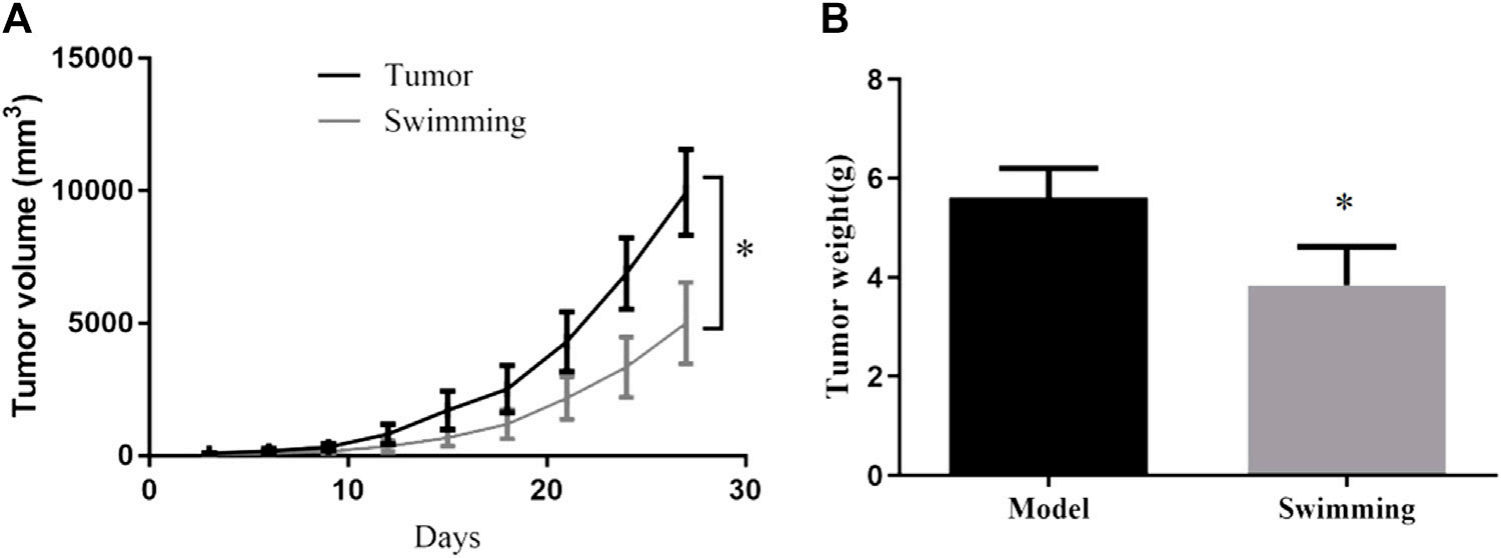
Effects of swimming on tumor volume and tumor weight of CT-26 bearing mice. **(A)** Tumor volume was monitored during the exercise period for 28 days. **(B)** Tumor weight was determined by electronic scale at the end of the experiment. Data are present as mean ± SD. *p < 0.05, *vs* Tumor.

To assess the benefits of swimming on tumor growth, CT-26 cells were transplanted into BALB/c mice and followed by quantitative daily swimming. Monitor of tumor volume indicated that swimming obviously alleviated tumor volume, when compared to Tumor group (Figure 1A; *p < 0.05 *vs*. Tumor group). Consistently, determination of tumor weight confirmed that swimming significantly attenuated tumor weight (Figure 1B; *p < 0.05, *vs*. Tumor group).

### Swimming Alleviates the Decrease of Muscle Function and Autonomic Activity of CT-26 Bearing Mice

**FIGURE 2:**
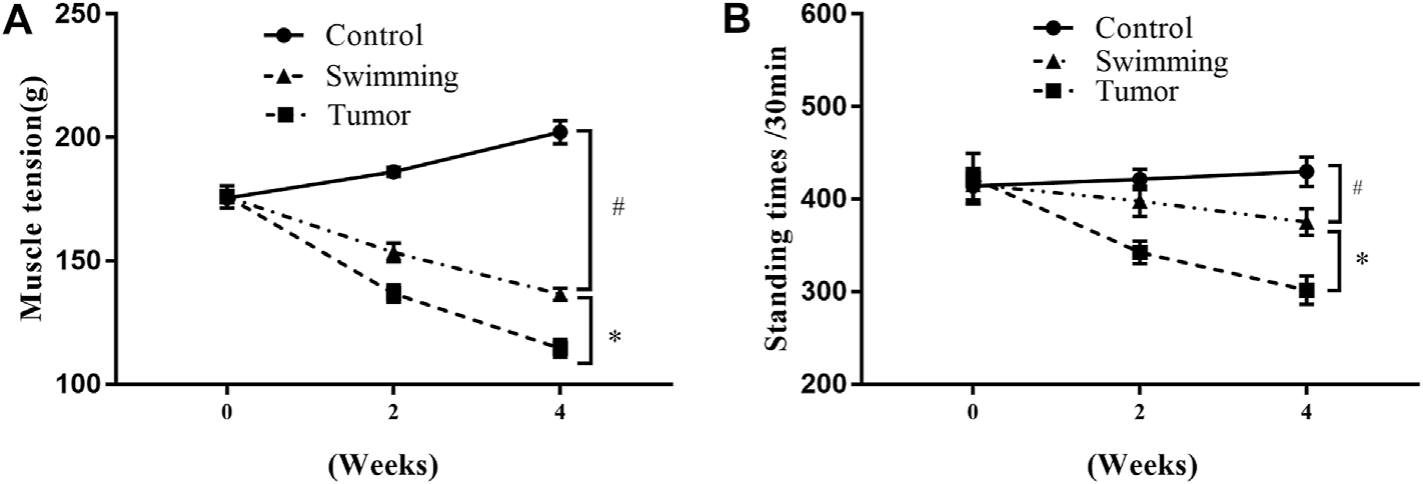
Effects of swimming on muscle function in transplanted tumor mice. **(A)** Skeletal muscular tension of mice was quantified by the grip-strength test. The strength test was performed before training, after the 2nd and 4th weeks of training. **(B)** Autonomic activity of mice was measured by the multifunctional mice independent activities recorder. The autonomous activity of the mice was recorded. Data are shown as mean ± SD. #*p* < 0.05, *vs*. Control; *p < 0.05, *vs*. Tumor.

Tumor growth was accompanied by a significant decrease in muscle tension in the Tumor group at the 2nd and 4th weeks (Figure 2A, ^#^ *p* < 0.05 vs. Controlgroup), which was attenuated in the Swimming group (Figure 2A, ^*^ *p* < 0.05 vs. Tumor group). Similarly, compared to the Tumor group, swimming significantly limited the decrease in autonomic activity at the 2nd and 4th weeks (Figure 2B, ^#^ *p* < 0.05 vs. Control group; ^*^ *p* < 0.05 vs. Tumor group).

### Swimming Attenuates Muscle Wasting and Pathological Ultrastructure of CT-26 Bearing Mice

**FIGURE 3:**
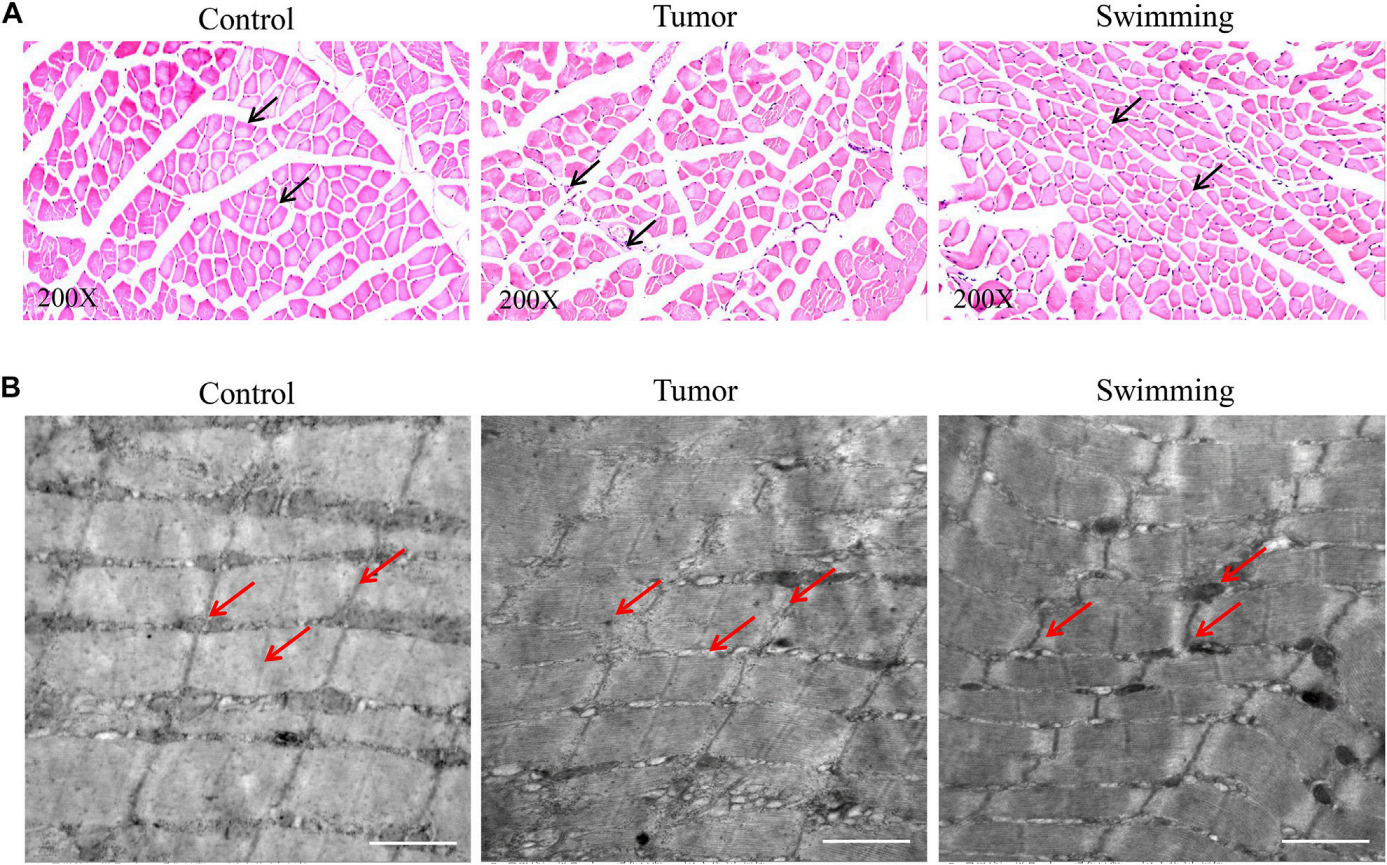
Microscopic observation of muscular tissues in Control, Swimming and Tumor groups. **(A)** HE staining was used to determine and observe the pathological changes of quadriceps muscles from each group. The images were taken at a magnification of ×200 **(B)** Transmission electron microscope was performed to determine and observe the microstructure changes of quadriceps muscles from each group.

Observation of pathological changes of quadriceps muscles by HE staining revealed a significant decrease of muscle fibers and cross sectional areas in the Tumor group suggesting a sign of muscle wasting, which were attenuated in Swimming group (Figure 3A). Observation of microscopic structural of muscle by transmission electron microscopy indicated a neatly arranged myofibrils of quadriceps with complete structure, distributedion of mitochondria near Z-line, and alternated light and dark bands of myofibrils with clearly I band and A band respectively (Figure 3B). However, we observed damaged or irregularly arranged myofibrils of quadriceps, with less or without clearly Z line and M line, as well as vacuolation and shrinkage of mitochondria in myofibrils of quadriceps in Tumor group, which were attenuated in Swimming group (Figure 3B).

### Swimming Reduced Cell Apoptosis, Decreased the Expression of Bax and Promoted the Expression of Bcl-2 in Quadriceps Muscle of CT-26 Bearing Mice

**FIGURE 4:**
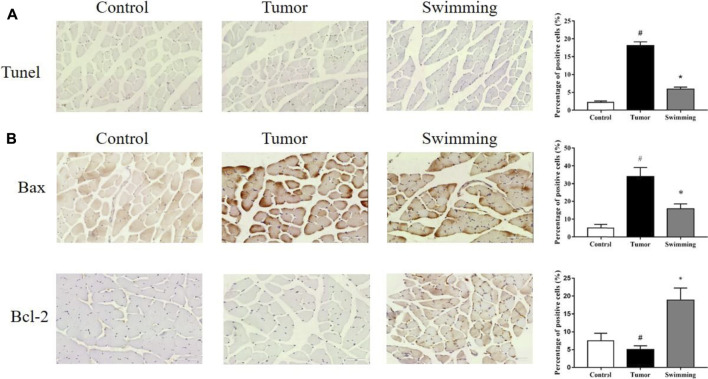
Effects of swimming on cell apoptosis and expression of Bax and Bcl-2 in muscle tissues. **(A)** TUNEL staining was performed determine the cell apoptosis in muscle tissues from each group. The images were taken at a magnification of ×400 by microscopy. The cells with brown nucleus were defined as apoptotic cells. **(B)** The protein levels of Bax and Bcl-2 in quadriceps muscle tissues of mice from each group was determined by IHC analysis. The images were taken at a magnification of ×400 by microscopy. The cells with brown staining were defined as positive stained cells. Data were presented as mean ± SD. #*p* < 0.05 *vs*. Control group; *p < 0.05 *vs*. Tumor group.

TUNEL analysis of quadriceps muscle tissues from each group revealed that the percentage of apoptotic quadriceps muscle cells was significantly increased in Tumor group, while were attenuated in Swimming group (Figure 4A, ^#^ *p* < 0.05 *vs*. Control group; *p < 0.05 *vs*. Tumor group). Moreover, further determination of Bax and Bcl-2 protein expression by IHC revealed that the expression level of pro-apoptotic protein Bax was decreased while that of anti-apoptotic Bcl-2 was obviously elevated in quadriceps muscle tissues of mice in Swimming group, which compared with Tumor group (Figure 4B, ^#^ *p* < 0.05 *vs* Control group; *p < 0.05 *vs*. Tumor group).

### Swimming Decreased the Levels of Inflammatory Cytokines in Quadriceps Muscle Tissues of CT-26 Bearing Mice

**FIGURE 5:**
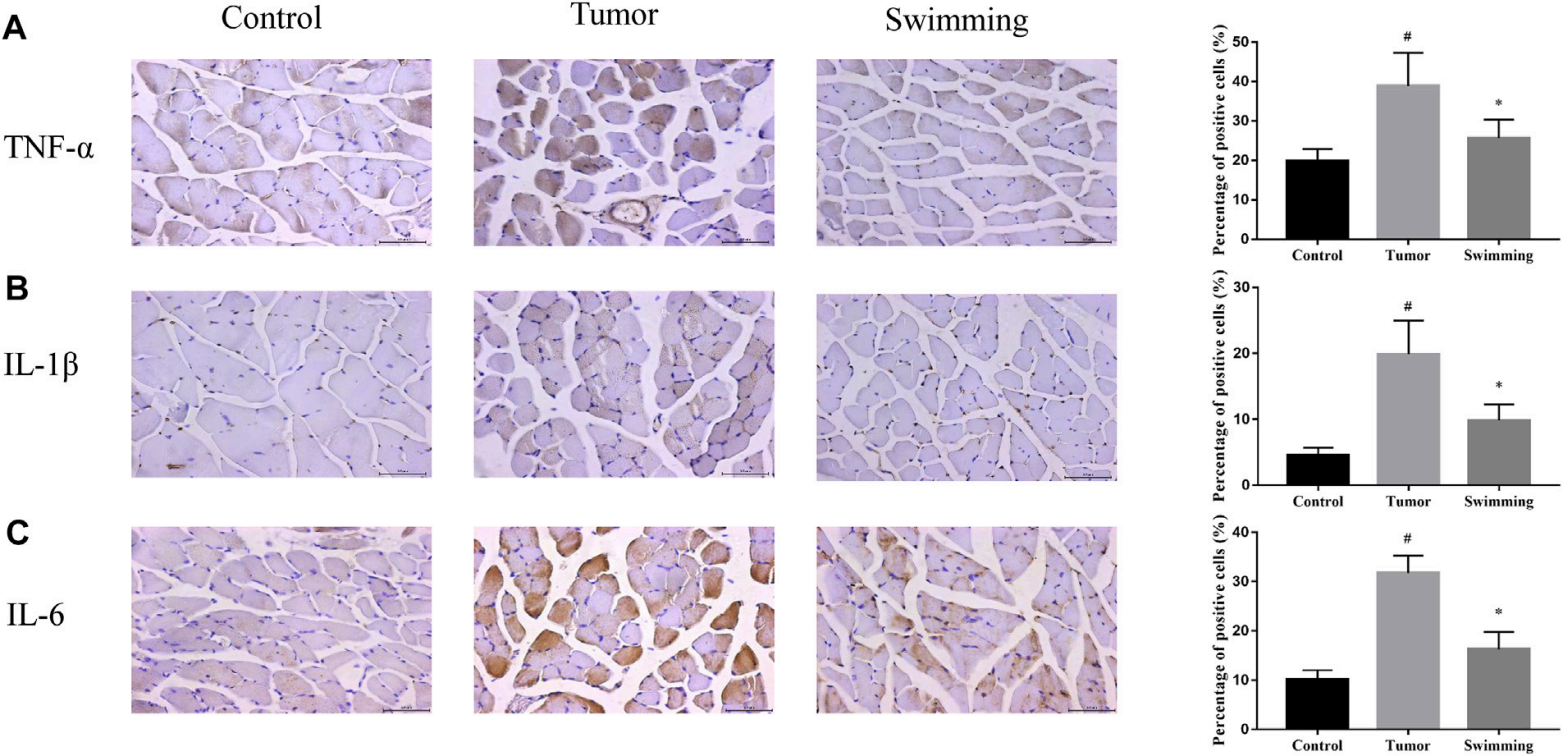
Effects of swimming on level of inflammatory cytokines in quadriceps muscle tissues. The protein levels of **(A)** TNF-α, **(B)** IL-1β and **(C)** IL-6 in quadriceps muscle tissues of mice from each group was determined by IHC analysis. The images were taken at a magnification of ×400 by microscopy. The cells with brown staining were defined as positive stained cells. Data were presented as mean ± SD. #*p* < 0.05 *vs*. Control group; *p < 0.05 *vs*. Tumor group.

To further explore the effects of swimming on attenuation muscle wasting and apoptosis, we further assessed the effects of swimming on level of inflammatory cytokines in quadriceps muscle tissues. As showed in Figure 5A, determination of inflammatory cytokines in muscle tissues from each group demonstrated that the level of IL-6 (Figure 5A, ^#^ *p* < 0.05 vs Control group) and TNF-α (Figure 5B, ^#^ *p* < 0.05 *vs*. Control group), as well as IL-1β (Figure 5C, ^#^ *p* < 0.05 *vs*. Control group) were obviously increased in the muscle of Tumor group, while were attenuated in that of Swimming group (Figures 5A–C; *p < 0.05 *vs*. Tumor group).

### Swimming Alleviated the Activation of NF-κB Signaling Pathway in Quadriceps Muscle Tissues of CT-26 Bearing Mice

**FIGURE 6:**
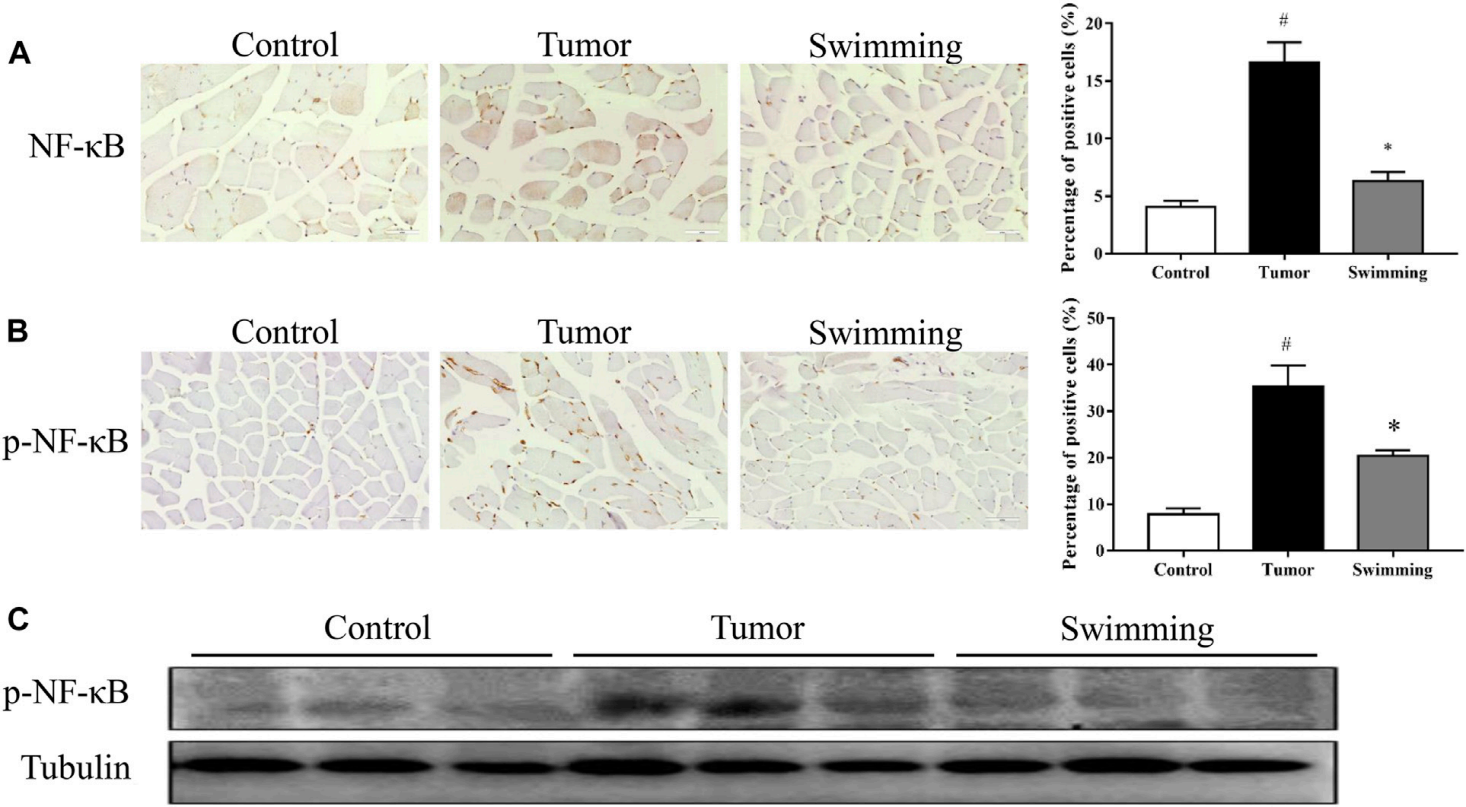
Effects of swimming on NF-κB signaling pathway. The protein levels of **(A)** NF-κB and **(B)** p-NF-κB in quadriceps muscle tissues of mice from each group was determined by IHC analysis. The images were taken at a magnification of ×400 by microscopy. The cells with brown staining were defined as positive stained cells. **(C)** The protein expression of p-NF-κB in quadriceps muscle tissues for each group was determined by Western-blot analysis. The tubulin was used as a loading control. Data were presented as mean ± SD. #*p* < 0.05 *vs*. Control group; *p < 0.05 *vs*. Tumor group.

To further explore the underlying mechanism of swimming on reducing the elevated levels of inflammatory cytokines, we further determined the activation of the NF-κB signaling pathway. As shown in Figures 6A–C. IHC analysis reveal that the levels of both NF-κB (Figure 6A) and p-NF-κB (Figure 6B) increased in quadriceps muscle tissues of mice in Tumor group (all ^#^ *p* < 0.05 *vs*. Control group), while all were attenuated in Swimming group (all *p < 0.05 *vs*. Tumor group). Consistently, Western-blotting analysis further confirmed that the protein expression of p-NF-κB significantly increased in the muscle tissues of Tumor group, which were decreased in that of the Swimming group (Figure 6C).

### Swimming Improves Quadriceps Muscle Metabolism of CT-26 Bearing Mice

**FIGURE 7:**
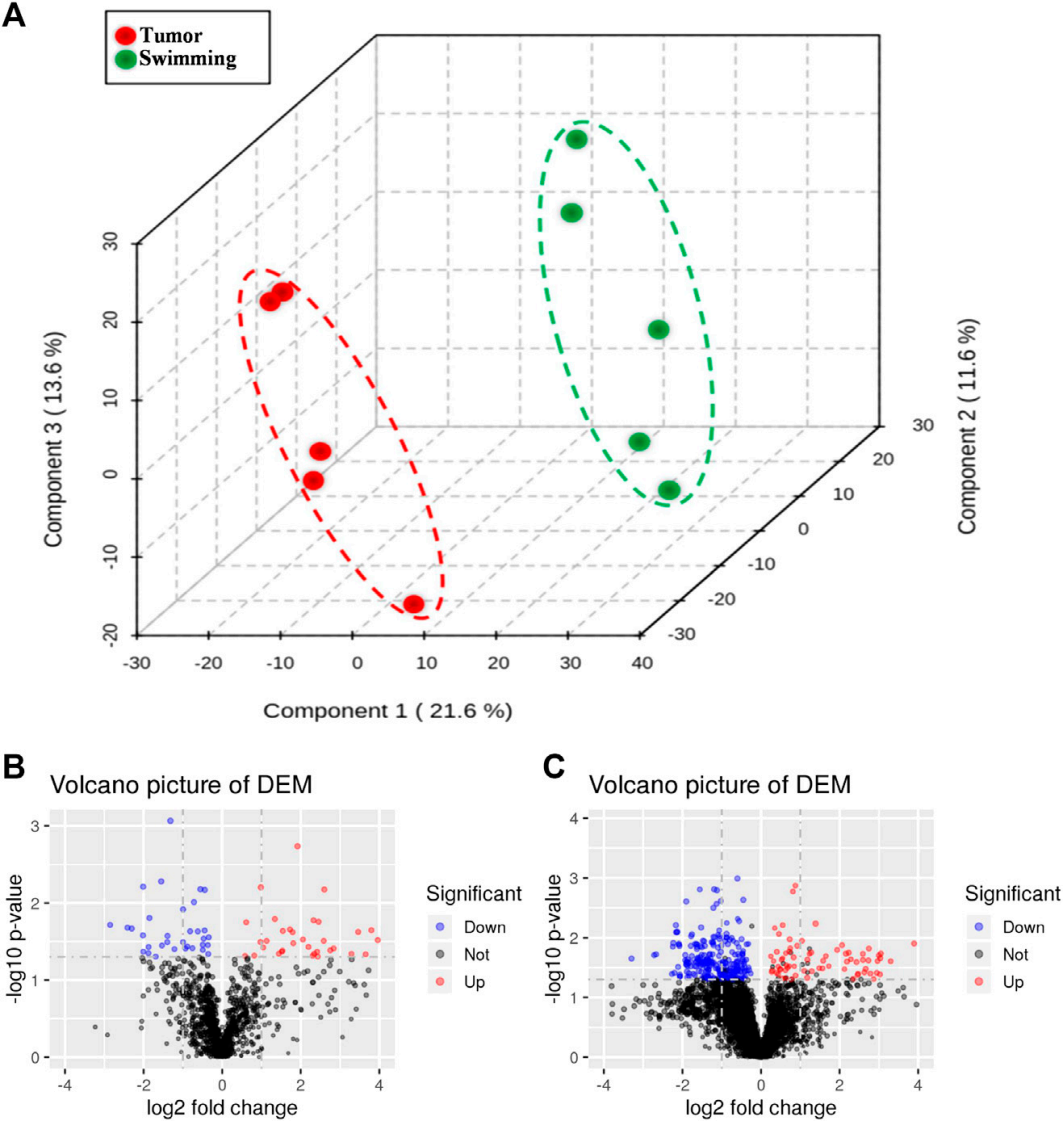
Changes in metabolic profiling after regular swimming training. **(A)** PCA plot was obtained by reduction of dimensionality for LC-MS data of Tumor group and Swimming group (explained variance by Component1 21.6%, Component2 11.6% and Component3 13.6%). The red circle shows the distribution of Tumor group in PCA plot, while the green one displays the location of Swimming group. Distance represents the difference between two groups. Volcano plots of differential metabolite screening between the Tumor group and the Swimming group at both **(B)** positive and **(C)** negative ion modes. The blue and red dots respectively mark down-regulated and up-regulated metabolites after swimming training, whereas the black ones represent no differences between the two groups.

**FIGURE 8:**
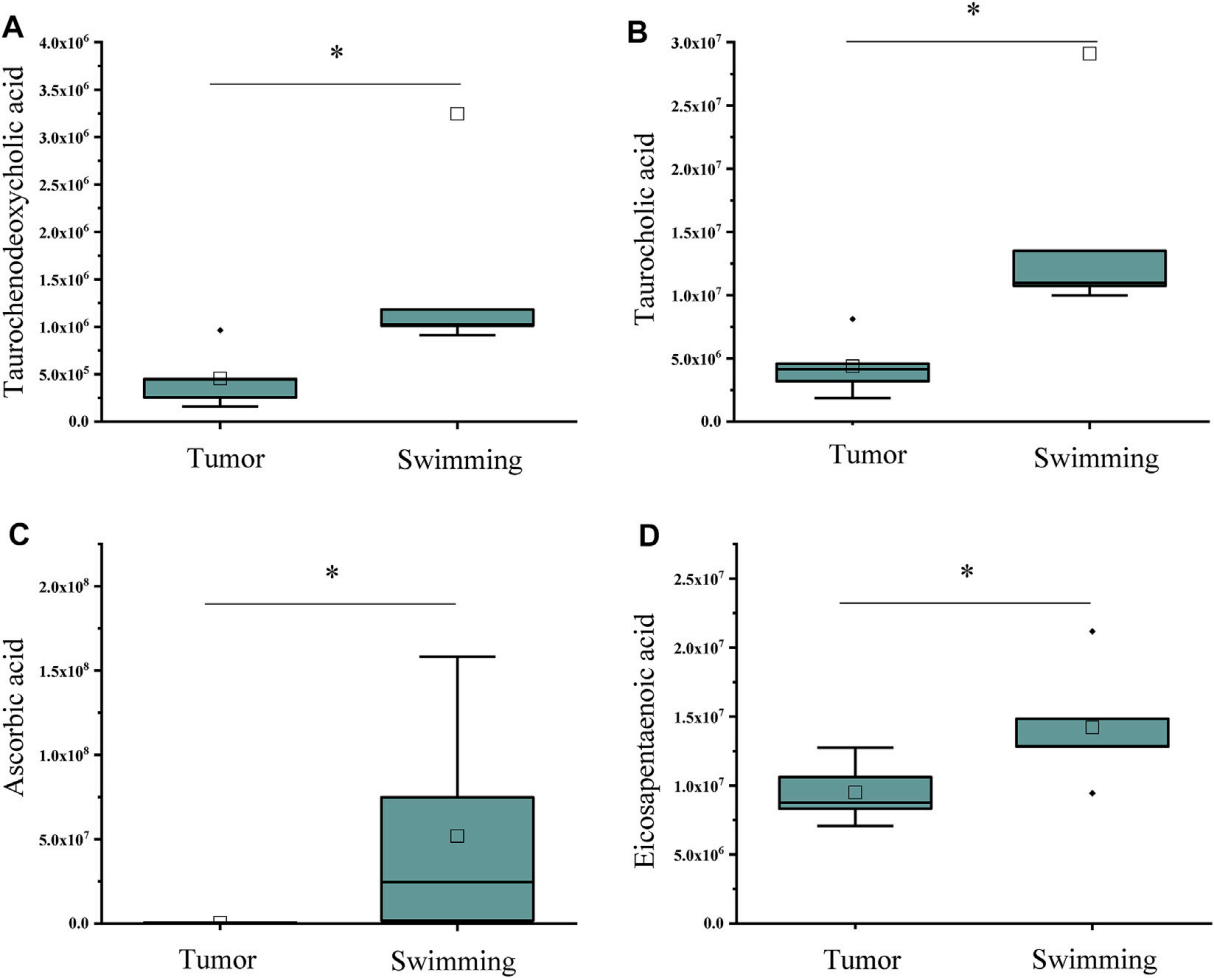
Statistical analysis of differential metabolites associated with inflammation and immune regulation. The levels of **(A)** Taurochenodeoxycholic acid, **(B)** Taurocholic acid, **(C)** Ascorbic acid and **(D)** Eicosapentaenoic acid in muscle tissues between Tumor and Swimming groups. *p < 0.05 vs the Tumor group.

Based on the benefits of swimming on preventing tumor growth and muscle wasting, we further determined the metabolomic profiling of quadriceps muscle obtained from mice of both Tumor and Swimming groups. PCA score plots clearly showed good separation between the two groups, indicating regular swim training makes a big difference in the metabolic profile of muscle (Figure 7A). The ions with variable importance in the projection (VIP) values > 1.0 were identified as the potential DEMs. Volcano plots exhibited the variation tendency of the metabolites with VIP values > 1.0 and adjusted by *p* values < 0.05 between two groups (Figure 7B). Compared with the Tumor group, a total of 76 DEMs were identified under anion mode (43 up-regulated and 33 down-regulated), and 330 were identified under the cationic mode (179 up-regulated and 151 down-regulated). Among the DEMs, the level of taurochenodeoxycholic acid, taurocholic acid, ascorbic acid and eicosapentaenoic acid in Swimming group were significantly higher compared with the Tumor group (Figure 8, all *p < 0.05 *vs*. Tumor group).

## Discussion

Muscle wasting in cancer cachexia contributes to resistance to treatment, low quality of life and death (Guo et al., 2017). We therefore explored the therapeutic approaches in an attempt to counteract cancer cachexia and improve treatment response, as well as quality of life of cancer patients. Using a tumor-bearing mice of CT-26 cells (Acharyya et al., 2005), the current study confirmed that swimming obviously attenuated the tumor growth, increased muscle strength and autonomic activity of CT-26 cells bearing mice. Moreover, swimming also alleviated muscle wasting, pathological ultrastructure of quadriceps muscles in CT-26 bearing mice. Mechanistic studies revealed that swimming reduced cell apoptosis and down-regulated the protein expression of Bax, up-regulated the protein expression of Bcl-2 in muscle tissues of CT-26 bearing mice, and inhibited the activation of NF-κB and its downstream pro-inflammatory cytokines (including TNF-α, IL-6 and IL-1β) which might be one of its underlying mechanisms. These studies suggest the potential of swimming as a supplementary therapeutic approach for cancer treatment.

More than 80% of patients with advanced cancer develop cancer-associated weight loss, a syndrome characterized by a forfeiture of muscle and a decline in functional status, quality of life, and survival (Doles et al., 2018). Physical activity has proved to be an effective therapeutic strategy due to its effect on both strength and muscle mass (Wang et al., 2021). Moderate exercise could relieve muscle wasting and prevent the loss of muscle strength through reducing the levels of reactive oxygen species (ROS), carbonylated proteins, markers of autophagy, and improving antioxidant capacity (Ballaro et al., 2019).

Due to the difficulty for cancer patients to perform high-intensity exercises, we assessed the effects of swimming (low-intensity exercises) on tumor growth and muscle wasting. As expected, swimming obviously alleviated tumor growth in CT-26 bearing mice, which is consistent with a previous study (Almeida et al., 2009). More importantly, swimming also attenuated the decrease of muscle strength and autonomic activity, as well as muscle fibers and cross-sectional areas of quadriceps muscles in CT-26 bearing mice. These studies suggest swimming might have contributed to the improvement in muscle wasting.

Consistently, TUNEL assay indicated that swimming significantly reduced the number of TUNEL positive staining cells in quadriceps muscles of CT-26 bearing mice. Usually, muscle wasting is accompanied by the apoptosis of a large number of muscle cells, corresponding expression changes of Bcl-2 and Bax also occur in muscle tissue (Murphy et al., 2011). Interestingly, mechanistic study revealed an up-regulation of Bcl-2 and down-regulation of Bax on protein levels in quadriceps muscles of CT-26 bearing mice after swimming, which might be one of the mechanisms of swimming on attenuating cell apoptosis in quadriceps muscles of CT-26 bearing mice. However, the complex mechanism should be further explored by determining the activation of related signaling pathway. Taken together, comparing with other voluntary exercise, swimming exhibits potential on serving as a therapeutic approach for cancer patients, particularly the elderly cancer patients, which might be more acceptable.

The therapeutic potential of swimming on cancer cachexia and muscle wasting encouraged us to further explore its underlying mechanism, due to the essential role of systemic inflammation on cancer induced muscle wasting and cachexia (Pin et al., 2015). Consistent with the previously study (Tisdale 2009), we observed the increase of inflammatory cytokines of TNF-α and IL-6 levels in quadriceps muscles of CT-26 bearing mice, which were significantly reduced after swimming. Increase of inflammation cytokines lead to the activation of transcription factor NF-κB, resulting in promoting muscle wasting and atrophy (Cai et al., 2004). More importantly, NF-κB pathway activation further promotes the synthesis of cytokines, contributing to muscle wasting as described above (Bonetto et al., 2016). Consistently, we found a significant increase of both NF-κB and p-NF-κB expression in quadriceps muscles of CT-26 bearing mice, which were significantly reduced after swimming. These results suggested that the suppression of NF-κB pathway activation and synthesis of inflammation cytokines might be one of the underlying mechanisms for swimming on attenuating cancer induced muscle wasting. However, the translocation of NF-κB should be further assessed both *in vivo* and/or *in vitro* systems.

With the development of metabolomics, metabolism is emerging as one of the key factors among various mechanisms that contribute to the regulation of the signaling pathways leading to apoptosis (Andersen and Kornbluth, 2013; Matsuura et al., 2016) and the development of inflammation in cancer (Gaber et al., 2017). Recently, the results acquired in both experimental and clinical studies clearly demonstrate that energy and protein dysmetabolism are closely related to muscle wasting during cancer cachexia (Penna et al., 2018). It is becoming increasingly evident that cachexia can be effectively improved by modulating muscle metabolism (Carson et al., 2016; Aversa et al., 2017; Penna et al., 2018).

Therefore, further metabolomic profiling analysis of quadriceps muscles between Swimming and Tumor groups identified a variety of DEMs, including taurochenodeoxycholic acid, taurocholic acid, ascorbic acid and eicosapentaenoic acid, which are closely related to inflammation and immune regulation. Among them, taurfodeoxycholic acid plays an important role in anti-inflammatory effects through the TGR5 receptor-induced cAMP-PKA-CREB signaling pathway, specifically by activating kappa light chain enhancer in B cells, reducing the activities of TNF-α, IL-1β and IL-6 (Qi et al., 2020). Taurocholic acid not only exhibits the effects of anti-inflammation, lowering blood pressure, and reducing the amplitude and frequency of cardiac contraction (Taranto et al., 2001), it is also involved in immune response by directly inhibiting the apoptosis of immune cells at different stages (Schwarz et al., 1975; Rodriguez-Garay et al., 1999). Ascorbic acid is a hexacarboxylate, also known as vitamin C, was involved in inhibiting the growth of HCT116 cells in mice, prolonging the survival rate of CRC patients, enhancing the sensitivity of colorectal cancer to chemotherapy, and reducing the adverse reactions of radiotherapy and chemotherapy (Siegel et al., 2014; Roncucci and Mariani, 2015). Eicosapentaenoic acid is an omega-3 polyunsaturated fatty acid, which has an effect on anti-tumor, anti-inflammatory, anti-oxidative stress, and reduces the risk of cardiovascular disease (Pappalardo et al., 2015; D'Eliseo and Velotti, 2016). In addition, it can also balance metabolism, inhibit proliferation and induce apoptosis (Brinton and Mason, 2017). Combined with our findings, we assumed that reducing the level of muscle inflammation by metabolic compensation in the Swimming group may also be one of the ways to attenuate muscle wasting in CT-26 bearing mice.

## Conclusion

Swimming maintains the muscle fine structure, attenuates muscle wasting, and improves muscle function, suggesting its potential in serving as a therapeutic approach for cancer patients. Suppression of NF-κB signaling pathway, reduction in the level of inflammatory factors, elevated levels of differential metabolites associated with anti-inflammatory and anti-apoptosis in quadriceps muscles of CT-26 bearing mice might be the important underlying mechanisms contributing to the benefits of swimming on attenuating muscle wasting.

## Data Availability

The original contributions presented in the study are included in the article/Supplementary Material, further inquiries can be directed to the corresponding authors.
