# Corrigendum: Swimming attenuates muscle wasting and mediates multiple signaling pathways and metabolites in CT-26 bearing mice

**DOI:** 10.3389/fmolb.2022.1005797

**Published:** 2022-09-02

**Authors:** Jiapeng Li, Qiurong Xie, Liya Liu, Ying Cheng, Yuying Han, Xiaoping Chen, Jia Lin, Zuanfang Li, Huixin Liu, Xiuli Zhang, Haichun Chen, Jun Peng, Aling Shen

**Affiliations:** ^1^ The Department of Physical Education, Fujian University of Traditional Chinese Medicine, Fuzhou, China; ^2^ School of Physical Education and Sport Science, Fujian Normal University, Fuzhou, China; ^3^ Academy of Integrative Medicine, Fuzhou, China; ^4^ Fujian Key Laboratory of Integrative Medicine in Geriatrics, Fuzhou, China; ^5^ Rehabilitation Industry Institute, Fujian University of Traditional Chinese Medicine, Fuzhou, China; ^6^ Provincial University Key Laboratory of Sport and Health Science, School of Physical Education and Sport Sciences, Fujian Normal University, Fuzhou, China

**Keywords:** swimming, colorectal cancer, muscle wasting, NF-κB, metabolite

In the published article, there was an error in **Affiliation 1**. Instead of “1The Department of Physical Education, Fuzhou, China,” it should be “1The Department of Physical Education, Fujian University of Traditional Chinese Medicine, Fuzhou, China.”

The authors apologize for this error and state that this does not change the scientific conclusions of the article in any way. The original article has been updated.

